# How to Treat Childhood Sexual Abuse Related PTSD Accompanied by Risky Sexual Behavior: A Case Study on the Use of Dialectical Behavior Therapy for Posttraumatic Stress Disorder (DBT-PTSD)

**DOI:** 10.1007/s40653-021-00421-6

**Published:** 2021-11-18

**Authors:** Regina Steil, Angelina Schneider, Laura Schwartzkopff

**Affiliations:** 1 Institute of Psychology, Department of Clinical Psychology and Psychotherapy, Goethe University, Frankfurt, Frankfurt, Germany; 2 Center for Mind, Brain and Behavior (CMBB), University of Marburg and Justus Liebig University, Giessen, Germany

**Keywords:** Childhood sexual abuse, Dialectical behavioral therapy, Posttraumatic stress disorder, PTSD, Risky sexual behavior

## Abstract

Childhood and adolescent sexual abuse (CSA) is a traumatic experience associated with a variety of short- and long-term negative consequences. Theoretical models assume that an abuse related and learned distorted image of sexuality might lead CSA survivors to feel obligated to provide sex or engage in unwanted sexual practices in order to gain affection or prevent abandonment. Dialectical behavioral therapy for posttraumatic stress disorder (DBT-PTSD) is tailored to people with PTSD and comorbid emotion regulation deficits. This case study presents the results of an outpatient DBT-PTSD treatment of an adult patient with posttraumatic stress disorder following sexual and physical abuse. DBT-PTSD was used to treat the patient’s complex psychopathological problems and to decrease her risky sexual behavior, which manifested itself in highly dangerous sexual practices with her partner. The treatment took place over a period of 18 months, with a total of 72 sessions. At the end of the treatment, the patient no longer met criteria for PTSD as indicated by large reductions in the assessments used. Furthermore, she managed to distance herself from risky sexual practices and to remain in a satisfying relationship.

## Theoretical and Research Basis for Treatment

Childhood and adolescent sexual abuse (CSA) has been associated with a variety of negative short- and long-term psychological consequences, such as posttraumatic stress disorder (PTSD), depression, anxiety, substance abuse and low self-esteem (Kendall-Tackett et al., 1993; Neumann et al., 1996; Paolucci et al., 2001). The probability of the development of PTSD is particularly high, with victims of sexual abuse showing a 5 to 8 times higher risk of developing PTSD compared to non-sexual abuse victims (Cutajar et al., 2010; Molnar et al., 2001; Walker et al., 2004). PTSD is characterized by four sets of symptom clusters: intrusive memories of the traumatic event, avoidance, hyperarousal, and negative alterations in mood or cognitions (American Psychiatric Association, 2013).

Aside from post-traumatic stress symptoms, victims of childhood and adolescent abuse are also effected in their later interpersonal and relationship functioning. Research assessing the impact of sexual abuse on adult sexual functioning indicates that sexual abuse survivors are not only likely to experience sexual dysfunctions related to desire, arousal, or orgasmic ability, but are also prone to sexual risk behaviors (Bornefeld-Ettmann et al., 2018; Stephenson et al., 2014). Risky sexual behaviors include engagement in unprotected sex, engaging in sex work, having sex with multiple partners or strangers, and engagement in punitive relationships involving sadomasochistic practices (Abajobir et al., 2017; Abrams & Stefan, 2012; Sandnabba et al., 2002; Senn et al., 2008). More recent research in the field of indirect self-harm in adolescents and young adults examines and understands risky sexual activities and intentional engagement in physically abusive relationships in the light of sex as self-injury (SASI), according to which dysfunctional sexual behavior is used to generate or regulate intense emotions (Jonsson et al., 2019; Zetterqvist et al., 2018). In a sample of 5743 Swedish students (*M* = 17.97 years, *SD* = 0.63) Zetterqvist et al. (2018) found that 2.2% (*n* = 125) of the adolescents had used sex as a means of self-injury. In these participants, the rate of traumatization and exposure to sexual abuse (48.8%) was higher than in participants who reported other forms of nonsuicidal self-injury (NSSI) (11.9%). Moreover, former victims of child and adolescent sexual abuse show an increased vulnerability to experience sexual revictimization. The risk of revictimization seems to be partially mediated by sexual self-esteem and sexual risk behaviors (Steil & Bornefeld-Ettmann, 2017; Van Bruggen et al., 2006). Research has found that emotional dysregulation can lead to significantly more revictimization events in survivors of abuse, with sexual risk behavior being an important link and risk factor (Messman-Moore et al., 2010).

Several theoretical models have been offered to explain the effects of CSA on adult sexual and relationship functioning. For a further understanding of the connection between sexual abuse and its implications for sexual and relationship functioning, the conceptualization of Finkelhor and Browne (1985) seems especially important. Finkelhor and Browne (1985) describe the consequences of sexual abuse based on four traumagenetic dynamics: traumatic sexualization, betrayal, powerlessness, and stigmatization. According to this model, abuse survivors not only experience sexuality in a developmentally inadequate and interpersonally dysfunctional way, but also adopt misconceptions about their self-worth, sexual morals, and power to control their lives. These distortions may persist into adulthood and negatively impact the sexual and relationship functioning of survivors (Finkelhor & Browne, 1985; Fortier et al., 2009). As a result, abuse survivors may feel obligated to provide sex in order to gain affection or attention in a relationship, or they might misunderstand and accept violence or other unwanted sexual practices by their later partners as a normal part of sexuality (Davis & Petretic-Jackson, 2000; Sandnabba et al., 2002).

Regarding the far-reaching psychological consequences of sexual abuse on victims and the associated impairments in relationship functioning, psychological treatment appears to be urgently required to help traumatized CSA survivors. Meta-analysis on the treatment of PTSD in adults reveal that especially trauma-focused psychological interventions such as cognitive and exposure-based therapy are effective and the first-line treatments for PTSD (Cusack et al., 2016; Watts et al., 2013). Research findings further suggest that interventions that take emotion regulation deficits into account, may further help to reduce rates of risky sexual behavior and subsequent risk for sexual revictimization (Messman-Moore et al., 2010).

Dialectical behavioral therapy for posttraumatic stress disorder (DBT-PTSD; Steil et al., 2011) is specifically tailored to individuals with severe PTSD following CSA and comorbid emotion regulation deficits. DBT-PTSD is based on classical DBT (Linehan, 1993), trauma-focused exposure and cognitive-based interventions, as well as innovative interventions, such as skills training to improve interpersonal functioning or cognitive and imagery interventions to improve the self-image (Steil et al., 2011; Steil et al., 2015). The treatment model is designed to enable patients to activate, endure and attenuate trauma-related and other painful emotions.

Thus, DBT-PTSD enables patients to relate their cognitive, emotional, and behavioral escape strategies in reaction to trauma associated stimuli, and to use DBT skills to reduce dysfunctional behavior and regulate emotions (Steil et al., 2011). Moreover, DBT-PTSD focuses on improving the patient´s negatively distorted self-concept. A more positive self-concept can not only have a positive effect on relationships and partnership, but also on sexual self-esteem and, through this, on overall satisfaction with sexuality (Bohus et al., 2019; Steil et al., 2011).

So far, the efficacy and acceptance of inpatient DBT-PTSD has been demonstrated in two trials, both with large effect sizes (Bohus et al., 2013; Steil et al., 2011). In the first randomized controlled trial (RCT), the efficacy of a 12-week residential DBT-PTSD program was compared to a usual treatment in a sample of 74 women (Bohus et al., 2013). Between-group effect sizes were large for all outcome measures, including the Clinician-Administered PTSD Scale (CAPS; Schnyder & Moergeli, 2002; *g* = 1.57), PDS (Griesel et al., 2006; *g* = 1.27) and Beck Depression Inventory-II (BDI-II, Beck et al., 1996; *g* = 1.13).

More recently, the effectiveness and feasibility of DBT-PTSD was evaluated in an outpatient treatment study that found large pre- to follow-up effect sizes for both PTSD symptoms (CAPS, d = 1.30) and borderline symptoms (BSL-23 (Bohus et al., 2009); d = 1.20) in a sample of 21 female PTSD patients after CSA (Steil et al., 2018). In a recently published large RCT on 193 outpatients suffering from PTSD and comorbid difficulties in emotion regulation, DBT-PTSD has shown large effect sizes and superiority in comparison to the state-of-the-art PTSD treatment Cognitive Processing Therapy (Bohus et al., 2020). The results of these two trials demonstrate the efficacy of DBT-PTSD in an inpatient treatment setting.

The central aim of this article is to present the use of DBT-PTSD to treat a woman diagnosed with PTSD as a clinical outpatient. Treatment goals include addressing complex PTSD symptoms, and also the reduction of sexual and relationship difficulties and dangerous sexual risk behaviors.

## Case Introduction

The case has been properly disguised so that identification of the patient will not be possible.

### Presenting Complaints

Ms. T. is a 31-year-old fashion designer, has no children and lives in a stable relationship with her partner, who is two years older than she. At the age of 19, she was imprisoned by her first boyfriend in his home for approximately two months, where she experienced sexual and physical abuse almost daily. As a result, she reported having frequent intrusive symptoms in the form of unwanted memories and flashbacks, as well as intense psychological stress and physiological reactions in the presence of trauma-related triggers (e.g. media containing violence). In retrospect, the post-traumatic symptoms existed since the index trauma had occurred. Even if the criteria of a borderline personality disorder were not fulfilled at the beginning of therapy, features seemed to have been more pronounced in her early adulthood. In the past, Ms. T. suffered from atypical anorexia, panic disorder with agoraphobia, and depressive disorder and, in her twenties, irregularly consumed various drugs (e.g., cannabis, ecstasy, LSD) and alcohol in the context of parties. Before starting the treatment, she had initial consultations with several therapists, but never had any outpatient or inpatient psychiatric or psychotherapeutic treatment.

During the initial interview, she reported that her partner's sadomasochistic tendencies, that is, his tendency of deriving sexual pleasure from inflicting pain and humiliation on her (i.e. by choking her or using clamps and shackles), are triggers for her PTSD symptoms. She managed her triggers through avoidance; additionally, she reported difficulties in remembering certain aspects of the trauma (e.g. the face of the perpetrator). Exploration in session also revealed feelings of guilt ("I could have walked out – why did I stay?"), hyperarousal, sleep and concentration difficulties, as well as nervousness. About twice a month, Ms. T. used nonsuicidal self-injury (i.e. with razor blades or by intense scratching) as a strategy to alleviate acute negative affect or affective arousal. Furthermore, she described interpersonal problems, especially in the relationship with her current partner. Ms. T. reported feeling small and insecure, and experiencing a great fear of being abandoned by her partner. She tried therefore to avoid "mistakes" in the relationship (“I thought I was only good for sex”). With the help of the treatment, she wanted to process what she had experienced, achieve symptom reduction, become emotionally stable, and learn how to deal with difficult relationship situations in a more functional way.

### History

Ms. T. is an only child and grew up with her parents. She described her father as very busy but also strict, giving her a feeling of “not being good enough”. He didn’t tolerate any mistakes, so that Ms. T. put herself under enormous pressure in order to gain his love and attention. Her mother, described as self-centered, always suffered from extreme mood swings and a high need for recognition. Furthermore, Ms. T. reported that the mother negatively influenced the relationship between her and her father by frequently devaluing the father and presenting herself as a "victim" during the frequent marital conflicts. In the past, her relationship with her mother was more like a friendship, but later Ms. T. realized that her mother had instrumentalized her for her emotional needs. The relationship between the parents was described as very conflict-ridden as a result of which Ms. T. experienced her home as a "hell", and constantly felt caught between her two parents.

Due to bullying in school, Ms. T. increasingly became a lonely adolescent. Neither in friendships nor in her family did she experience stable relationships, so that her first abusive boyfriend, whom she met at the age of 18 in a chat room, found, as she said, "fertile ground". He initially wooed her strongly in order to force her to move out of her parents' house. After moving in with him, Ms. T. faced two months of emotional, sexual, and physical abuse. The perpetrator later became mentally conspicuous in public and was admitted to an inpatient facility. In response to the events, Ms. T. developed clinically significant PTSD symptoms, intermittent symptoms of anorexia nervosa, and later also depressive symptoms. Moreover, at the age of 19 she attempted suicide with an overdose of medication and alcohol. In her twenties, family burdens increased when her parents divorced, and her mother became addicted to alcohol. At this time, she occasionally used drugs and alcohol at parties and was once raped at a party while intoxicated.

After final secondary-school examinations, the patient completed a commercial apprenticeship and worked in an accounting office for four years. Due to her dissatisfaction, she decided to follow her dream and use her longtime passion for fashion by successfully completing an apprenticeship as a seamstress in order to then become self-employed with her own small business. She had been with her current, and for the first time loving and supportive partner for one year before treatment started. However, her partner experienced sexual pleasure mainly through dominance and devotion, and the couple used various instruments during sadomasochistic sex (a cage, shackles, clamps). Ms. T. was also regularly choked and strangled by her partner during sexual interaction, sometimes resulting in her losing consciousness.

Although Ms. T. liked to take the submissive role, the extreme practices of her partner did not correspond to her preferences. In a willingness to give her partner “what he needs", she either submitted to the practices, despite a high level of discomfort and distress, or avoided sexual contact.

## Assessment

Based on the SKID-I (First et al., 1996; Wittchen et al., 1997) and -II, Clinician-Administered PTSD Scale (CAPS; Blake et al., 1995: Schnyder & Moergeli, 2002) and established self-assessment procedures, Ms. T. was diagnosed with PTSD. In view of the self-injuring behavior, the unstable self-image and the affective instability, borderline personality disorder was considered in the differential diagnosis, although the criteria were not fully met according to the SKID-II (First et al., 1997; Fydrich et al., 1997). The Brief Symptom Inventory (BSI; Derogatis, 1993; Franke, 2000) indicated a clinically relevant symptomatology with increased scores on the scales of somatization, compulsivity, insecurity in social contact, anxiety, aggressiveness, and phobic fear. The initial score of 30 on the Posttraumatic Stress Disorder Checklist for DSM-5 (PCL-5; Krüger-Gottschalk et al., 2017; Weathers et al., 2013) indicated the presence of PTSD, with lower values for the criterion C (avoidance). At the time of the initial assessment, her Beck Depression Inventory II (BDI-II; Beck et al., 1996; Hautzinger et al., 2006) score was 17, indicating mild depressive symptoms. A table of the results can be found at the end of the manuscript.

## Case Conceptualization

In accordance with the underlying concept of DBT-PTSD (Bohus et al., 2013; Steil et al., 2011), a hierarchical, structured approach based on the following therapy goals was planned:

1. Development of a sustainable therapeutic relationship and motivation for change.
2. Promotion of an understanding of the origin and maintenance of the patient´s PTSD symptoms.
3. Clarification and, if necessary, reduction of harmful sexual behavior.
4. Reduction of problem behaviors and emotional instability.
5. Reduction of PTSD symptoms.
6. Identification and modification of dysfunctional cognitions.
7. Improvement of self-esteem and self-care.
8. Improvement of work situation and pursuit of individual life goals.
9. Stabilization of therapeutic achievements and relapse prevention

## Course of Treatment, Assessment of Progress, and Complicating Factors

The treatment took place over a period of 18 months, with a total of 72 sessions (weekly sessions of 50 minutes each, with exposure sessions up to 200 minutes).

### Assessment, Therapeutic Relationship, and Motivation for Change

The focus of the first sessions was the establishment of a stable therapeutic relationship with an empathic and validating approach. In view of a one-time suicide attempt and a one-time verbal suicide threat directed at the partner in a high-stress situation at the beginning of therapy, a non-suicide contract was formulated and signed and a very elaborate safety plan including the daily assessment of suicidal thoughts was developed. In addition to a detailed analysis of former experiences, effects of earlier relationship experiences on the current experience and behavior, and possible transference to the therapeutic relationship were analyzed (McCullough, 2000). Because DBT-PTSD aims at supporting the patient to live a fulfilled life despite the traumatic experience, the “old way” (living with the symptoms) and the “new way” (e.g. use of skills instead of escape strategies) was discussed to clarify values and goals. Moreover, dysfunctional cognitions and emotions as well as symptom maintaining behaviors were analyzed.

### Psychoeducation and Treatment Model

To promote the client's understanding of PTSD and explain the therapeutic rationale, psychoeducation on PTSD and its development, as well as the establishment of an individual case formulation model were carried out at the beginning. An individual disorder model was collaboratively developed, considering predisposing factors and reactions to trauma, such as trigger stimuli and escape and avoidance behaviors. The maintaining function of escape and avoidance strategies was further emphasized by developing a cognitive model. The patient responded very gratefully to the psychoeducation and reported a sense of relief through a better understanding of her disorder.

### Clarification and Reduction of Harmful Sexual Behavior and Couple Conversations

In view of the risky sexual practices, the sexuality of the couple and possible associated risks for the patient were explored in detail. In particular, the choking and strangulation, which the patient said she experienced as satisfactory, was discussed in terms of medical and physical dangers such as brain damage from frequent lack of oxygen, or even death. At first, she reacted skeptically, almost in disbelief, and adopting a different point of view was difficult for her. Through the constant problematization and the open communication of the therapist´s concerns about the patient - among other things in the context of a joint appointment with the supervisor which is compulsory in DBT-PTSD - the patient began to question the practices used by the couple. The patient was instructed to talk to a neurologist and find out what happens to her brain when she is choked on a regular basis. Ms. T. began to observe how she felt when carrying out the various sexual practices, and so managed to differentiate between practices that were pleasant and those that were less pleasant for her. In a couple talk at the beginning of the therapy, relationship problems could be named and ways of solving them could be developed (e.g. the use of code words). As a result, Ms. T. was increasingly able to speak openly with her partner about their sexuality, to clearly set personal boundaries and to communicate her own needs and desires, but also give up dangerous sexual practices that potentially involved bodily harm, such as strangulation. All in all, the couple managed to fulfill their mutual sexual needs in a balanced way. The partner proved to be very supportive and caring during the therapy. Initially and throughout therapy, the couple communicated openly in order to promote the partner's understanding of the problem and to explain the initial problems of boundaries, communication and emotional dysregulation. In addition, the couple's conversations enabled open communication regarding the couple's relationship problems that had arisen because of the patients’ symptoms. In this way, solution strategies for difficult situations could be worked out together (e.g. communication of the current state of tension). With the loving support of her partner, the patient was able to learn functional strategies for regulating emotions and resolving conflicts by realizing the needs of both partners.

### Establishing Skills for Emotion Regulation and Reduction of Problem Behavior

Within the DBT-PTSD dynamic hierarchy of treatment foci, PTSD was not targeted until life-threatening and therapy-interfering behaviors were under control (mainly self-harm and dissociation). Over the course of treatment, the patient completed a diary card to monitor symptoms and problematic as well as helpful behaviors. In addition, behavioral analyses were carried out to understand factors that influence self-harm and risky sexual behavior. Mindfulness exercises were used regularly at the beginning and the end of the session. The patient showed a great deal of interest in mindfulness and therefore practiced a lot on her own by using apps and meditational instructions. Over time the exercises were alternately instructed by the patient or the therapist. To promote the recognition of emotions and to increase distress tolerance, emotion regulation skills (Bohus & Wolf-Arehult, 2013) were taught. Within a few weeks, the patient was able to give up her problem behavior and perceive, analyze, and regulate her own thoughts and feelings more attentively. Independently, Ms. T. also worked through the interpersonal skills and self-esteem modules that were previously discussed in the sessions (Bohus & Wolf-Arehult, 2013), for example she sought social feedback on her strengths or performed role plays asserting her needs.

### Processing of Guilt and Shame

The patient's feeling of guilt was processed with cognitive techniques by first exploring the patient's self-accusations. It became clear that Ms. T. overestimated her options for action at the time when she was imprisoned by her first partner ("I could have left the situation"; "I could have reported him to the police"). Trauma-related guilt was discussed using a pie chart. By means of a precise analysis of the processes and actions during the traumatic event and the use of helpful questions (e.g. "Why did you behave like this in that moment, and not differently?"), dysfunctional cognitions were questioned using socratic dialogue. The “hindsight bias” was discussed and the patient was able to rationally distance herself from her guilt. According to the emotional network model (Bohus & Wolf-Arehult, 2013), shame arising during exposure was attenuated by a change in the patient´s posture to one that is opposite of a posture of shame (e.g. putting as many body parts as possible in contact with the wall when standing close to it, resulting in a “proud” posture, and seeking eye contact with the therapist) and by cognitive impulses (e.g. "Who should actually be ashamed?").

### Preparation of Skills-assisted Exposure and Exposure Phase

The patient´s fears and concerns regarding the exposure phase were explored and questioned using socratic dialogue, thus helping the patient to distance herself well from her fears. The exposure took place in a four-week intensive treatment phase, with initially two double sessions per week. Therapist and patient defined the index trauma– the experience which currently causes the most distress. This memory (first sexual abuse) was reported verbally by the patient in the past tense with her eyes open. She then prepared a detailed, emotionally activating written report, confronting herself with the index trauma as homework and during the session. Subsequently, an in sensu activation of memory by means of intermittent activation of the trauma network (including the presentation of trauma-associated stimuli) and of the reference to the present (including the use of skills) the processing of hot spots took place. In between sessions, the patient was asked to listen to audiotape of the most recent session once a day. The patient quickly experienced a reduction in distress. Subsequently, a total of three different traumatic memories were processed in this way. The patient learned to apply the technique very reliably on her own.

During and after exposure exercises, the corresponding cognitions, emotions and impulses as well as the degree of distress were assessed to achieve a balance between activation of trauma-associated emotions and reference to the present. Dysfunctional cognitions and beliefs were modified using newly acquired detailed knowledge of the traumatic event. As a result, Ms. T. reported a significant reduction in post-traumatic symptoms.

At the end of the exposure phase, she stated that she was "rather bored" by the audiotapes, and that she was able to accept the traumatic memories as part of her past.

### Promotion of Self-esteem and Self-care

In addition to the continuous promotion of a benevolent attitude through mindfulness exercises, diary cards, and independent skills training, interventions to promote self-esteem and self-care were used. As an example, 30 positive characteristics of the patient were identified, which she then summarized in a laudatory speech for herself. Ms. T. stated that she had distanced herself from dysfunctional beliefs about herself formulated at the beginning of the therapy and tried not to let her actions be influenced by them. On the behavioral level, she also managed to reduce her workload, accept less productive days, and plan self-care activities.

### Consolidation of Treatment Success and Relapse Prevention

Towards the end of treatment, therapist and patient identified positive changes on the emotional, cognitive, and behavioral level. The problem of revictimization was addressed by providing information about its frequent occurrence and making the patient aware of warning signs of toxic partners, whereby she was able to recognize the perpetrator in almost all items and clearly identify her current partner as non-toxic, in spite of his sadistic sexual preferences. Ms. T. wrote down her individual treatment history, in the context of which she reflected on the development and maintenance of symptoms, learning successes and helpful strategies, possible future risk situations and future plans. In a final evaluation of her therapy goals, the patient stated she had achieved her goals in almost all areas.

Table 1 illustrates the course of the patient´s symptoms of PTSD and comorbid problems.

**Table 1:** Symptom change over the course of the DBT-PTSD with Ms. T

	**Pre-treatment**	**During treatment**	**Post-treatment**
**CAPS-5-total**	**40**	**-**	**2**
**PCL-5**	**30**	**11**	**2**
**BSL-95**	**0.76**	**-**	**0.19**
**BDI-II**	**17**	**8**	**0**
**BSI**	**72**	**50**	**42**

*BDI-II* Beck Depression Inventory II (Becket al., 1996), *BSI* Brief Symptom Inventory (Derogatis, 1993), *BSL-95* Boderline Symptom List (Bohus et al., 2001), *CAPS-5* Clinican-Administered PTSD Scale for DSM-V (Schnyder & Moergeli, 2002), *PCL-5* Posttraumatic Stress Disorder Checklist for DSM-5 (Weathers et al., 2013), All values represent raw, nonstandardized scores

## Summary and Treatment Implications

This case study illustrates that experiencing childhood sexual abuse can have a long-term impact on different areas of mental health and can be associated with engaging in dysfunctional and risky sexual behaviors later in life. It also illustrates that DBT-PTSD can successfully be used to treat not only PTSD and comorbid problems after sexual abuse, but also dysfunctional behaviors, such as risky sexual behaviors. The treatment was carried out over a period of 18 months with a total of 72 sessions, which took place weekly, with sessions towards the end of therapy at intervals of up to four weeks. In view of the patient´s initial problem behavior, techniques for emotion regulation (above all behavioral analyses, diary cards, skills training) were applied before the trauma-focused work. Cognitive interventions were used to change dysfunctional beliefs about the trauma and its consequences, especially guilt, shame, and low self-esteem. In the preparation of the exposure phase, upcoming fears were addressed. A significant reduction in PTSD symptoms was achieved over a four-week intensive trauma exposure phase. A self-care and value-oriented approach was encouraged. Overall, a reorientation through the radical acceptance of the trauma which had been experienced and the regaining of a positive self-image was achieved. At the end of the treatment, the patient experienced a significantly improved relationship with her partner - also from the partner's point of view - by dealing functionally with conflicts and criticism, and by discussing the needs of both partners. For the entire therapeutic team of therapist, supervisor, and patient, it was amazing, that the romantic relationship was not impaired but improved by stopping the dangerous choking and strangulating.

In sum, the use of DBT-PTSD decreased the client’s risky sexual behaviors by the skills she developed and the change in self-appraisal and self-concept, allowing her a new awareness of her own needs and sexual identity. By the resolution of the trauma and as she learned to question her distorted thinking and beliefs, the patient no longer needed to use sexuality as a means to avoid abandonment, nor did she further need to avoid sexual contact.

Regarding the application of DBT-PTSD for clinicians and researchers, we particularly emphasize the importance of an accurate sexual history, and exploration of sexually risky practices, as well as sexual dysfunction, to also enable the patient to speak openly about respective topics. The therapist’s offer to explore and take into account a patient’s dangerous sexual practices should, however, always respect the patient's autonomy. Furthermore, inviting the partner to couple sessions is a useful complement to individual sessions, providing greater insight into the relationship dynamics.

In conclusion, this case study shows for the first time the feasibility of using DBT-PTSD to treat not only PTSD but also risky sexual behaviors in a woman with a history of sexual abuse and current risky sexual behaviors. The results encourage further research focusing on this kind of risk taking in patients suffering from PTSD after childhood abuse and further research on the effects of DBT-PTSD on these kinds of accompanying symptoms.
